# Bullying in Adolescents Practising Sport: A Structural Model Approach

**DOI:** 10.3390/ijerph192013438

**Published:** 2022-10-18

**Authors:** José Manuel Ortiz-Marcos, Ana Lendínez-Turón, Miguel Ángel Solano-Sánchez, María Tomé-Fernández

**Affiliations:** 1 Department of Developmental and Educational Psychology, Faculty of Education and Sports Sciences (Melilla Campus), University of Granada, 52005 Melilla, Spain; jm.ortiz.marcos@ugr.es (J.M.O.-M.); analendinez@ugr.es (A.L.-T.); 2 Department of Applied Economics, Faculty of Social and Legal Sciences (Melilla Campus), University of Granada, 52005 Melilla, Spain; 3 Department of Research Methods and Diagnosis in Education, Faculty of Education and Sports Sciences (Melilla Campus), University of Granada, 52005 Melilla, Spain; mariatf@ugr.es

**Keywords:** bullying victim, bullying aggressor, adolescents, sport, structural model

## Abstract

This article aims to analyse the relationship between the bullying aggressor and bullying victim profile related to practising or not practising sport in adolescents living in southern Spain. The research includes male and female participants aged between 12 and 16 years in different secondary schools in the provinces of Andalusia, Ceuta and Melilla in the period between February 2022 and June 2022. The study aims to extend the existing scientific, theoretical and empirical knowledge on the influence of playing sport or not on disruptive bullying attitudes in adolescents. To this end, two initial hypotheses were designed; the first hypothesises that bullying victim behaviours are associated with future bullying aggressor behaviours when practising sport; and the second states that victim behaviours are associated with future bullying aggressor behaviours when not practising sport. To verify them, SPSS software was used for the preliminary analysis of the scale and sociodemographic profile. Additionally, the study is based on structural equation modelling methodology and variance-based methods employing SmartPLS v3.3 software. The results show the importance of sport or physical activity to reduce the chances of carrying out bullying actions on other peers and/or classmates. Therefore, it is considered necessary to prevent bullying in the classroom by implementing sports intervention programmes in educational centres.

## 1. Introduction

School climate and safety are at the forefront of current educational issues. Bullying is a fragment of the school climate and safety puzzle. Bullying, either as a victim or as an aggressor, can have a lasting impact on students [1]. Such a situation aims to generate discomfort in the victim, who is unable to defend himself/herself [2]. Along the same lines, [3] understand it as a prolonged behaviour of verbal insults, social rejection, psychological physical aggression and/or intimidation by some students towards others, in which the victim is exposed to repetitive negative behaviours developed by one or several aggressors in a helplessness statement. In other words, bullying is understood as the intentional action of consciously harming or being cruel to the victims. In general, traditional forms of bullying can be divided into three categories of aggression: physical (hitting, kicking), verbal (name-calling, teasing) and social (ignoring and isolating). Behind these behaviours lies an inequality of power (physical, verbal, psychological or social) between a weak victim and a strong aggressor that leaves the victim defenseless [4].

As is reflected by [5], bullying is one of the subsets of social aggression that started to be analysed more extensively 40 years ago and, in the last decade, attracted increasing interest from researchers in various countries, which is still ongoing to this day [6]. In this context, bullying is relevant as it is produced in the educational environment, where its consequences can have a significant impact [7]. This type of disruptive behaviour continues in the 21st century after decades of trying to improve school behaviour and coexistence. Consequently, it is necessary to consider its implications for future generations [8]. These disruptive behaviours are still not disappearing, despite the efforts of the educational community, and continue to increase through new forms of bullying [9]. In this sense, it is necessary to assess the state of affairs to find the current causes that provoke this behaviour so that academic and social authorities can work together to find an early solution [10]. These disruptive behaviours not only affect students during their compulsory education stage but can also transcend to the university or adult stage [11]. Moreover, due to technological advances, these problems caused in the school context are transferred to virtual spaces [12]. In fact, studies have found a strong relationship between the two types of bullying, observing that many victims/perpetrators of traditional bullying were also victims/perpetrators of cyberbullying [13]. In this sense, Buelga [14] has suggested that cybervictimisation and cyberbullying are part of a general pattern of violence, where using electronic devices is another way of bullying the peer group.

For this reason, researchers and education professionals must understand and comprehend the prevalence of school bullying to become agents of change within their school systems [15]. Authors such as [16] define bullying as “unwanted and aggressive behaviour among school-aged children that involves a real or perceived power imbalance that is repeated, or has the potential to be repeated, over time”. This definition is one of the most widely used by experts in the field [17].

The main concern shown by experts studying school bullying, such as education professionals, is the myriad of possible effects it can have on the victim and the aggressor [18]. Some research has indicated that both victims and bullies are at increased risk of suicidal ideation and suicide attempts [19,20]. Other adverse effects may include poor mental health, depression and social maladjustment [21]; poor academic performance [22]; and illicit drug use and substance abuse for victims [21,23]. Refs. [6,24] have linked an environment of physical education (PE) to bullying acts. In this sense, it has been suggested that victims of school bullying tend to avoid school contexts that make them feel vulnerable, among which the authors highlight those found in physical activities [25].

However, some prevention programmes have considered the physical education factor in the role against violence in schools due to its valuable effects by favouring the externalisation of emotions and social skills improvement [26]. In this sense, it should be noted that among healthy lifestyle habits, (non-competitive) sports practice promotes responsibility and improves coexistence [27], being an excellent means of transmitting values [28] and promoting prosocial attitudes [29]. Thus, it can be applied as a means of prevention and treatment of school bullying and victimisation and carries a lower risk of developing aggressive behaviours and deviant behaviours [30]. Authors such as [31] and [32] indicate that physical activity, and more specifically sport, can have a positive impact on the body, mind, empathy and cooperation among peer groups. Therefore, the teachers of PE’s importance in fostering a positive environment during lessons has been highlighted, favouring students’ empowerment and social empathy development [33]. Following [34,35], sports coaches and PE teachers should not only help students to improve their physical fitness but also develop their social skills, improving their empowerment and growth in learning to live constructively in society [36].

Instead, other studies highlight the need and relevance of the proactive and, therefore, the non-reactive role of physical education teachers in dealing with bullying [37], thus highlighting the importance of physical education teachers assessing the environment in which classes take place to promote inclusive environments that do not encourage violence among young people. Along these lines, [38] and [39] propose various steps to develop a bullying-free environment in physical education: the previous assessment of bullying types and their frequency in physical education lessons (e.g., by surveys collecting students’ and teachers’ perceptions); the elaboration of a curriculum that vindicates proactive actions (e.g., by integrating activities requiring more collaboration and cooperation, rather than competition); and its measurement (e.g., by asking teachers, parents and students, about perceived variations in bullying experiences).

Therefore, this research aims to analyse the relationship between the bullying aggressor (BAgres) and bullying victim (BVic) profile related to practising or not practising sport in adolescents. The study aims to extend the existing scientific, theoretical and empirical knowledge on the influence of playing sport or not on disruptive bullying attitudes in people aged between 12 and 16 years.

## 2. Materials and Methods

**Figure 1:**
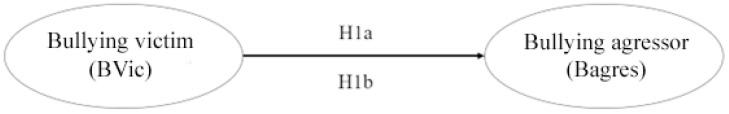
Proposed model.

As a result of the above, the following hypotheses are presented (the overall proposed model in Figure 1):

**H1a.**  *bullying victim (BVic) behaviours are associated with future bullying aggressor (BAgres) behaviours when playing sport.*

**H1b.**  *bullying victim (BVic) behaviours are associated with future bullying aggressor (BAgres) behaviours when not playing sport.*

### 2.1. Participants

Non-probability purposive sampling is used. The number of participants analysed (N = 1454) in the research is considered eligible. The number obtained is representative of the total number of adolescents stipulated (*n* = 944,570) from the southern and northern African Spanish centres of secondary education. This gives a confidence level of 95% and a maximum estimation error of 5% [40]. The sample selection consisted of 1454 adolescents from Andalusia and the North African cities of Ceuta and Melilla. Participants ranged in age from 12 to 16 years (M = 13.98; SD = 1.353), reflecting a similar representation of 725 males (49.9%) and 729 females (50.1%). The selected sample is taken from the different provinces of southern Spain, such as the autonomous cities Ceuta and Melilla to carry out the research, as they are the Spanish areas where the highest cases of bullying among adolescents are manifested [41,42]. The distribution of participants by province is shown in Table 1, by practising (or not) sport in Table 2.

### 2.2. Sample and Instrument Design

The questionnaire is divided into three blocks. The first addressed questions relating to the sociodemographic profile of the sample, such as gender, age, whether or not they had been physically active, the city where they were studying, as well as their religion, ethnicity and race. These questions were polytomous. Second, questions related to victim bullying behaviours, and third, questions regarding aggressor bullying behaviours. In these last two blocks, the questions were formulated through five-point Likert scales, being 1 not at all, 3 enough and 5 a lot. Block two, which referred to questions concerning victim bullying, consisted of 14 questions, while the third block was composed of 15 questions concerning bullying harassment.

The questionnaire was administered in educational centres in each of the provincial capitals of Andalusia and in the autonomous cities of Ceuta and Melilla, in a period ranging from February to June 2022, to obtain a total of 1632 questionnaires. However, after an initial filtering process, the final number of valid questionnaires was 1454. The questionnaire is elaborated, taking as the basis the Spanish version of the European Cyberbullying Intervention Project Questionnaire (ECIPQ) [43,44], the European Bullying Intervention Project Questionnaire [45], Hall’s Cyberbullying questionnaire [46] and Bullying in Sport Questionnaire (BSQ), which were designed to measure the frequency of bullying in sport [47].

The adapted instrument is designed for adolescents between 12 and 16 years old and is structured with 52 items that evaluate three blocks (sociodemographic variables, victims and aggressors). Following theoretical indications [48], the elaboration of the questionnaire is carried out in three phases: preliminary, exploratory and final, which determined the 29 definitive items of the instrument. This questionnaire focused mainly on the variable if the students had been physically active or not and the influence it exerts on bullying.

### 2.3. Preliminary Reliability Analysis and Statistical Analysis

To carry out the proposed analysis, different software is employed. First, the SPSS programme is used for data preparation and to carry out the preliminary analysis of the scale and sociodemographic profile. To test the reliability of the scale used, a Cronbach’s alpha test value of 0.959 is obtained. The last is clearly above the minimum required value of 0.7, as indicated by reference authors in the field [49].

On the other hand, the study was based on structural equation modelling (SEM) methodology, specifically on variance-based methods (V-SEM), employing SmartPLS v3.3, a tool widely used in the field of social sciences [50,51,52]. The use of partial least squares methodology has several advantages, such as the fact that it does not assume any specific distribution [53] or that it can estimate structural models with small sample sizes [54,55].

## 3. Results

### 3.1. Sociodemographic Profile and Questions Raised

The mean sociodemographic profile of the sample selected corresponds to a female who is physically active, aged 13/14 years, whose religion is Christianity and of Castilian ethnicity and white race. Table 3 shows a more detailed breakdown of the sociodemographic results. Table 4 shows the descriptive statistics of the items answered by the study participants: mean and standard deviation.

### 3.2. Measurement Model Assessment

The measurement evaluation of the model is carried out through an individual analysis, where factor loadings are addressed and, at the level of internal consistency, through the Dijkstra-Henseler composite reliability, convergent validity through the Average Variance Extracted (AVE) and discriminant validity through the Heterotrait-Monotrait ratio (HT-MT ratio). For the existence of individual validity, factor loadings must be greater than 0.70 [56], although other authors such as [57] point out that in the initial stages of research, this minimum value must be 0.6. Regarding internal consistency, the Dijkstra-Henseler ρA) composite reliability values should be above 0.70, 0.5 for AVE and HT-MT ratio values below 0.85 [58]. Table 5 present the reliability and validity analysis of the measurement model for both the YES-SPORT and the NO-SPORT models. The obtained data show an optimal validity of the measurement model, both at the individual level (it was necessary to remove indicators that did not exceed the minimum loading threshold for both the YES-SPORT and the NO-SPORT models) and at the level of internal consistency, as indicated by the mean extracted variance and the Heterotrait-Monotrait ratio (Table 6).

### 3.3. Structural Model

**Figure 2:**
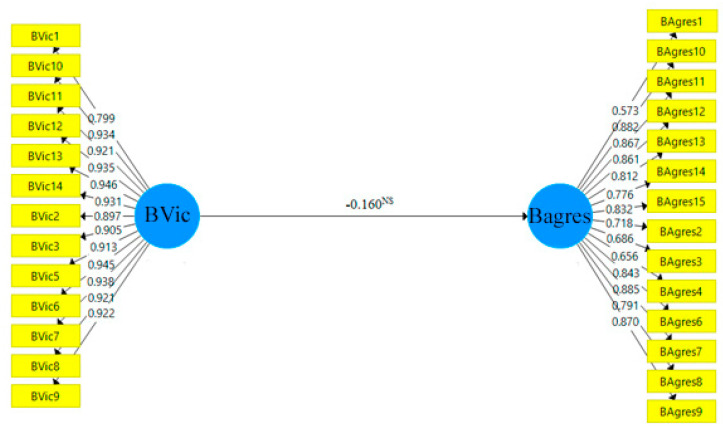
YES−SPORT Final Structural Model. Note: NS = not supported.

**Figure 3:**
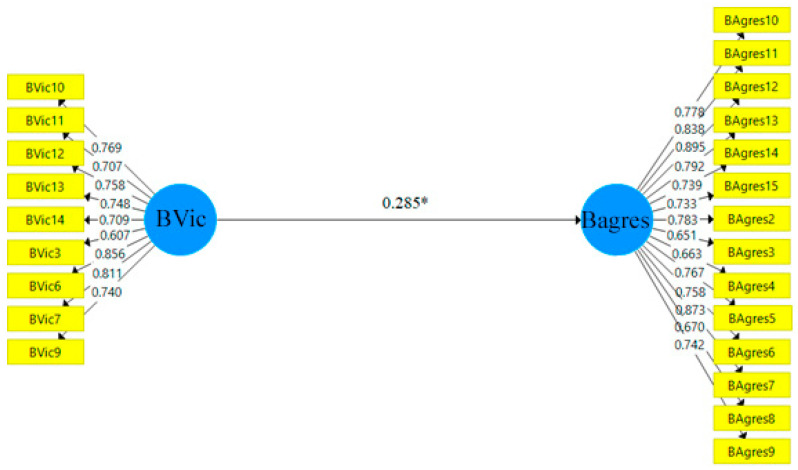
Final structural model NO-SPORT. Note: * *p* < 0.05.

Based on Section 3.2, and to test the significance of the hypotheses presented (Section 2), a bootstrapping of 10,000 subsamples [59] is developed, obtaining Student’s t and the associated *p*-value, as well as the confidence intervals, thus carrying out hypothesis testing from a parametric and non-parametric perspective. Table 7 shows the results obtained. Thus, the final structural models for each group are presented in Figure 2 (H1a) and Figure 3 (H1b).

## 4. Discussion

The results obtained show differences in bullying behaviours when they have been previously bullied. Thus, the first hypothesis (H1a), which stated that victim bullying behaviours are associated with future aggressor bullying behaviours when practising sport, has not been supported, indicating that if a sport is practised, this behaviour is not repeated. The results are associated with those obtained in previous studies [27] in which it is highlighted that, among healthy lifestyle habits, the practice of sport promotes responsibility and improves coexistence among the peer group. Along the same lines, authors such as [26] reflect that prevention programmes have considered the role of sport as an intervention against school violence since its effects are beneficial, as they favour the externalisation of emotions and improve social skills.

Sports practice becomes an excellent means for the transmission of values [28] and a promoter of prosocial attitudes [29]. Therefore, it can be applied as a means of prevention and treatment of school bullying and victimisation and carries a lower risk of developing aggressive behaviours and deviant behaviours [30]. School bullying, within this context, should be taken with particular relevance, as it should be taken into consideration that it occurs in the educational environment, where its consequences can have a more significant impact on the physical, psychological and social development of the youngest [7]. Despite preventive interventions, this type of disruptive behaviour is still present today, and after decades of trying to improve school behaviour and coexistence, it is necessary to consider the consequences it may have for future generations [8]. Along the same lines, other studies indicate that these disruptive behaviours have not disappeared, despite the efforts of the educational community, and continue to increase through new forms of bullying [9]. For this reason, and as demonstrated [60], the practice of physical-sports activities in young people and adolescents provides beneficial effects on their physical, psychological and social development. In addition, although bullying can occur in different contexts, it has been observed that the incidence of bullying in sport is lower than that observed in the school context, where it presents rates with wide ranges of incidence ranging from 10% to 48% [61]. This theory is reinforced by [62], who argued that many adolescents spend most of their free time in sports environments, which allows them to interrelate with their colleagues and fellows, offering them the opportunity to develop physically and socially, and establishing and reinforcing interpersonal relationships between students.

A study applied in schools with a high prevalence of bullying [63], showed that students who participated in a sports programme to act against bullying improved their knowledge and attitudes against bullying, and as students, after completing the programme, reported an improvement in their knowledge about bullying and a reduction in bullying behaviours, victimisation and fighting compared to students who did not participate in the study.

On the other hand, the second hypothesis (H1b), the one that hypothesised that victim behaviours are associated with future bullying aggressor behaviours when not practising sport has been supported, indicating that when not practising sport or physical activity, there is a tendency to carry out future bullying aggressor behaviours when having been a victim of aggression in the past [64]. As reflected in the literature, these behaviours must be intervened upon to avoid “the spiral of violence”, where they not only occur in the adolescent stage but can transcend to the adult stage or other higher educational stages [11]. The technological context implies that these disruptive behaviours are transferred to virtual spaces [12]. In fact, some studies show a strong relationship between both types of bullying, noting that many victims/perpetrators of traditional bullying were also victims/perpetrators of cyberbullying [13].

Evidence suggests that when a child or adolescent spends a significant amount of time in sedentary behaviours or activities, it is associated with poorer health and attitudinal outcomes [28]. According to [65], it may be directly related to the development of antisocial behaviour and aggression [66] and more negative feelings [67]. Therefore, it is important to understand how the bullying situation is related to young people’s compliance or non-compliance with physical activity recommendations and sedentary behaviour [68]. Similarly, these findings can be supported by the results of [69] where it is highlighted that young people who reported that they had bullied others or had been bullied were associated with less sport practices. However, students who reported more sporting activity contributed significantly to predicting that this type of activity decreased bullying levels among adolescents [70]. Considering previous studies, the results highlight the importance of focusing on beliefs about sport practice as an intervention strategy to improve interpersonal relationships in adolescents involved in bullying [71,72,73].

## 5. Conclusions

The conclusions obtained highlight and confirm the two initial hypotheses put forward in the study, indicating the importance of sport or physical activity not only in terms of a better physical situation for the individual but also, as verified in the results of the study, in reducing the possibilities of bullying other classmates and/or fellows. Therefore, it is considered necessary, as a means of preventing this type of bullying, for schools to implement sports intervention programmes for this purpose in the classroom. In the present study, it seemed to be effective in reducing victimisation in school bullying and, as in many cases, in cyberbullying, as well as aggression in bullying, although with a more cautious interpretation. Teachers could apply this type of intervention, together with specialist teachers and programmes adapted to PE content, to reduce levels of bullying while working on aspects related to students’ socio-emotional skills.

Thus, it is considered fundamental for educational institutions to raise these educational intervention programmes from the different contents of the curriculum to include training in values and prosocial aspects of learning through sport, which will encourage empathy, autonomy and help among the peer group. In this sense, pedagogical models already proven in the scientific literature could be a solution, as with, for example, Cooperative Learning, the Personal and Social Responsibility Model or interventions based on dialogical learning in schools that are transformed into learning communities. These models or their combination could reduce the negative behaviours of schoolchildren in the adolescent stage, such as aggression and the effects of victimisation, contributing to solving coexistence problems and developing a positive climate for the personal development of adolescents.

As limitations of the study, it is necessary to consider with caution the data obtained in the research, since they do not constitute a sample of the total Spanish educational context—which is only characteristic of a Southern Spain context—, nor can they be extrapolated to European contexts. Despite these limitations, the sample is considered distinctive for the south of the country, which provides a solid approach to the phenomenon of bullying and how sports practice influences this phenomenon. Moreover, this research used a quantitative methodology, which may provide limited information about the construct. For this reason, it is recommended to extend the study to research that accumulates qualitative data to provide complementary information to the current findings and to contract them.

Despite all these limitations, the results obtained in the research process are worthy and accurate for the existing scientific vacuum of research carried out in Spain on this type of bullying and how the practice of sport helps to reduce this type of disruptive behaviour in adolescents, as bullying is on the rise, specifically in educational environments, especially in those with a large cultural influx.

As a result of these limitations, it is suggested that future research related to this study replicates the analysis of the model presented with other larger samples, using longitudinal designs, measuring at two or three different moments that would provide us with more accurate data on the analysed construct. On the other hand, another type of analysis is also proposed for future studies that address this aspect from the aforementioned methodological approaches. Therefore, it is recommended that future research ad-dresses all these limitations and study similar models with experimental, qualitative or mixed methodologies, also considering other theoretical perspectives based on modern human sciences and beyond educational theories to easily interpret some of the considerable empirical research carried out recently in many countries.

## Figures and Tables

**Table 1:** Distribution of participants by city.

City	Participants’ No.	%
Granada	107	7.4
Málaga	131	9.0
Almería	111	7.6
Jaén	161	11.1
Córdoba	193	13.3
Sevilla	205	14.1
Cádiz	117	8.0
Huelva	89	6.1
Melilla	172	11.8
Ceuta	168	11.6
Total	1454	100

**Table 2:** Participants practising or not practising sport.

Practising Sport	Participants	%
No	143	9.8
Yes	1311	90.2
Total	1454	100

**Table 3:** Sociodemographic profile.

Variable	%	Variable	%	Variable	%
Gender Male Female	49.9 50.1	Physical activity Yes No	90.2 9.8	Religion Christianity Judaism Islam Taoism Buddhism Other	70.2 5.4 4.1 1.1 0.3 18.8
Age 12 years old 13 years old 14 years old 15 years old 16 years old	16.7 23.8 23.2 17.6 18.7	City Granada Málaga Almería Jaén Córdoba Seville Cádiz Huelva Melilla Ceuta	7.4 9.0 7.6 11.1 13.3 14.1 8.0 6.1 11.8 11.6	Ethnicity Castilian Mongolian Gypsy Armenian Celtic Other	65.7 13.5 3.2 1.2 0.6 16.0
Race White Indigenous African Asian Nordic Other	37.3 20.8 4.4 1.4 1.0 5.2				

**Table 4:** Descriptive statistics of the items.

Item Code	Question	Mean	Std. Dev.
BVic1	Someone has called me names or insulted me because I have a different skin colour.	1.46	1.027
BVic2	Someone has called me names or insulted me because I am of a different ethnicity or religion.	1.41	0.945
BVic3	Made racist comments about my race, ethnicity or religion.	1.48	1.026
BVic4	Someone has told other classmates lies about my race, ethnicity or religion.	1.39	0.947
BVic5	I have been threatened because of my religious or ethnic tradition.	1.39	0.964
BVic6	They have promoted fear about the customs of my country or my ethnicity or religion.	1.41	1.001
BVic7	Someone has made fun of my family and me because of our traditions and customs.	1.42	1.017
BVic8	Someone has humiliated me because of my race.	1.41	1.014
BVic9	My group’s religious or ethnic activity has been ridiculed.	1.41	1.006
BVic10	I have been excluded or ignored because I am of a different race, religion or ethnicity from my peers.	1.39	0.983
BVic11	I have been hit for being different from them (skin colour, hair, dress, tradition, religion).	1.39	0.986
BVic12	They imitated my speech to make fun of my language.	1.37	0.949
BVic13	I have been told that my race, religion or ethnicity should be exterminated.	1.42	1.010
BVic14	I have been made fun of for wearing a different dress or garment from my peers.	1.38	0.984
BAgres1	I have called someone a bad word or insulted another classmate for having a different skin colour.	1.40	0.959
BAgres2	I have spoken abusive words or insulted other classmates because they are of a different ethnicity or religion.	1.39	0.985
BAgres3	I have made racist comments about other races, ethnicities or religions.	1.49	1.007
BAgres4	I have told other colleagues lies about other races, ethnicities or religions.	1.36	0.976
BAgres5	I have threatened other religious or ethnic traditions through messages.	1.17	0.608
BAgres6	I have attempted to create hatred towards other racial, ethnic or religious groups.	1.13	0.532
BAgres7	I have ridiculed religious or ethnic traditions other than my own.	1.16	0.608
BAgres8	I have mocked the traditions and customs of other families.	1.14	0.595
BAgres9	I have humiliated the religious or ethnic traditions of other groups.	1.15	0.626
BAgres10	I have ridiculed the religious or ethnic activity of another group.	1.12	0.579
BAgres11	I have excluded or ignored others because they are of a different race or belong to a different religion or ethnicity from the rest of my classmates.	1.11	0.554
BAgres12	I have hit classmates because they are different from me (skin colour, hair, clothing, tradition, religion).	1.14	0.981
BAgres13	I have imitated my partner’s speech to make fun of his or her language.	1.12	0.573
BAgres14	I have threatened that the race, religion or ethnicity of other classmates should be exterminated.	1.13	0.590
BAgres15	I have made fun of other classmates for wearing a different dress or garment than the rest of my classmates.	1.13	0.585

**Table 5:** Reliability and validity analysis of the measurement model (YES-SPORT Model).

YES-SPORT MODEL				NON-SPORT MODEL			
	Loads	_A	A.V.E.		Loads	_A	A.V.E.
Bullying victim (BVic)		0.952	0.606	Bullying victim (BVic)		0.919	0.559
BVic1	0.704			BVic3	0.607		
BVic2	0.769			BVic6	0.856		
BVic3	0.772			BVic7	0.811		
BVic5	0.750			BVic9	0.740		
BVic6	0.805			BVic10	0.769		
BVic7	0.858			BVic11	0.707		
BVic8	0.868			BVic12	0.758		
BVic9	0.856			BVic13	0.748		
BVic10	0.817			BVic14	0.709		
BVic11	0.769			Bullying aggressor (BAgres)		0.952	0.587
BVic12	0.760			BAgres2	0.783		
BVic13	0.665			BAgres3	0.651		
BVic14	0.694			BAgres4	0.663		
Bullying aggressor (BAgres)		0.963	0.655	BAgres5	0.737		
BAgres1	0.735			BAgres6	0.758		
BAgres2	0.707			BAgres7	0.873		
BAgres3	0.776			BAgres8	0.670		
BAgres4	0.689			BAgres9	0.742		
BAgres6	0.796			BAgres10	0.778		
BAgres7	0.903			BAgres11	0.838		
BAgres8	0.781			BAgres12	0.895		
BAgres9	0.902			BAgres13	0.792		
BAgres10	0.944			BAgres14	0.739		
BAgres11	0.733			BAgres15	0.733		
BAgres12	0.873						
BAgres13	0.810						
BAgres14	0.691						
BAgres15	0.931						

**Table 6:** Discriminant validity. Heterotrait-Monotrait Ratio.

		Bullying Aggressor (BAgres)
**Bullying Victim (BVic)**	YES-SPORT	[0.094; 0.248]
	NO-SPORT	[0.130; 0.470]

Notes: Brackets are the 95% confidence interval (two-tailed) with 5000 resamples-Practice sport (YES-SPORT) and No practice sport (NO-SPORT).

**Table 7:** Hypothesis testing.

		Original Sample	*t*	*p*-Value	95% CI	
					2.5%	97.5%
H1a	BVic → BAgres	−0.160 ^NS^	0.799	0.424	−0.256	0.436
H1b	BVic → BAgres	0.285 *	2.490	0.013	0.219	0.653

Notes: * *p* < 0.05; (10,000; two-tailed); t (9999; 0.05) = 1.96; t (9999; 0.01) = 2.57; t (9999; 0.001) = 3.29. NS = not supported.
